# Tumour spectrum in non-BRCA hereditary breast cancer families in Sweden

**DOI:** 10.1186/s13053-015-0036-z

**Published:** 2015-06-16

**Authors:** Camilla Wendt, Annika Lindblom, Brita Arver, Anna von Wachenfeldt, Sara Margolin

**Affiliations:** 1 Department of Oncology-Pathology, Karolinska Institutet Solna, S-17177 Stockholm, Sweden; 2 Department of Oncology, Karolinska University Hospital Solna, S-17177 Stockholm, Sweden; 3 Department of Clinical Genetics, Karolinska University Hospital Solna, S-17177 Stockholm, Sweden; 4 Department of Molecular Medicine and Surgery, Karolinska Institutet Solna, S-17177 Stockholm, Sweden

**Keywords:** Breast cancer, Endometrial cancer, Family history, Cancer syndrome

## Abstract

**Background:**

Approximately 30 % of all breast cancer is at least partly attributed to hereditary factors. Familial breast cancer is often inherited in the context of cancer syndromes. The most commonly mutated genes are BRCA1 and BRCA2 in hereditary breast and ovarian cancer syndrome. The genetic background in families with hereditary breast cancer without predisposing germ line mutations in BRCA1 and BRCA2 (non-BRCA families) is still to a large extent unclear even though progress has been made. The aim of this study was to compare cancer proportions in familial non-BRCA hereditary breast cancer compared to the general population in search of putative new breast cancer syndromes.

**Methods:**

Pedigrees from 334 non-BRCA hereditary breast cancer families in the county of Stockholm, Sweden, were investigated and the distribution of cancer diagnoses other than breast cancer was compared with the distribution of cancer diagnoses in the general Swedish population in two reference years, 1970 and 2010. A cancer diagnosis was regarded as overrepresented in the non-BRCA families if the confidence interval was above both population reference values.

**Results:**

We found that endometrial cancer was overrepresented in the non-BRCA families with a 6.36 % proportion (CI 4.67–8.2) compared to the proportion in the general population in the reference years 1970 (3.07 %) and 2010 (2.64 %). Moreover tumours of the ovary, liver, pancreas and prostate were overrepresented.

**Conclusion:**

In conclusion, we found an overrepresentation of endometrial cancer in our cohort of hereditary non-BRCA families. Our result supports previous inconsistent reports of a putative breast and endometrial cancer syndrome. An association has been suggested in studies of families with several cases of breast cancer in close relatives or bilateral breast cancer. To clarify this issue we suggest further studies on a breast and endometrial cancer syndrome in cohorts with a strong pattern of hereditary breast cancer. Identifying new breast cancer syndromes is of importance to improve genetic counselling for women at risk and a first step towards detection of new susceptibility genes.

## Introduction

Although breast cancer is the most common form of cancer in women, the incidence varies greatly, being highest in developed countries, but also increasing in developing nations. The standardised incidence in Western Europe is as high as 90 per 100,000 and approximately 10 % of all Swedish women will be affected by this disease [1, 2].

Identified risk factors include use of exogenous hormones, reproductive factors and obesity though a family history of breast cancer is the most important one. The risk increases with the number of close relatives affected, especially if they are young at the time of diagnosis. The age of onset in these families is lower than in the case of sporadic breast cancer [3, 4]. Depending on the number of affected relatives and the age at onset, the conferred risk ranges from almost doubled to fivefold compared to the 10 % population risk [3]. A Scandinavian twin study has shown genetic susceptibility in 27 % of all breast cancers [5].

Major advances in the understanding of breast cancer susceptibility were made in the 1990s when the two major high-risk breast cancer and ovarian cancer predisposition genes BRCA1 and BRCA2 were identified [6–8]. Significant for all identified high-risk breast cancer predisposition genes is that they are observed in the context of breast cancer syndromes involving not only breast cancer but also an increased risk of other tumour types. Apart from the Hereditary Breast and Ovarian Cancer Syndrome caused by mutations in BRCA1 and BRCA2, these include Li-Fraumeni Syndrome (TP53) [9], Cowden Syndrome (PTEN) [10], Peutz-Jeghers Syndrome (STK11) [11, 12] and Hereditary Diffuse Gastric and Lobular Breast Cancer Syndrome (CDH1) [13]. In addition, most of the identified moderate penetrance breast cancer genes also predispose to other tumour types. Besides an intermediate increased risk of breast cancer, CHEK2 mutations have been associated with an increased risk of bladder, colorectal, prostate and kidney cancer [14–16]. Mutations in BRIP1 have been associated with increased risk of breast and ovarian cancer [17, 18]. Carriers of deleterious PALB2 mutations have a moderate to high risk of breast cancer and also an increased risk of pancreatic cancer and ovarian cancer [19–21]. Inherited deleterious ATM mutations have been associated with both breast cancer and pancreatic cancer predisposition [22, 23]. These moderate risk genes confer a 2–4 fold risk compared to the 10 % risk in the general population. In recent years genome-wide association studies in large cohorts have identified multiple low risk variants that each confer a modest risk though the combined effect can be substantial [24].

Although deleterious BRCA1 and BRCA2 mutations constitute the most common breast cancer syndrome, they explain the genetic background in only 1–3 % in sporadic breast cancer populations and about 15 % of familial breast cancer [25–27]. Altogether, BRCA1, BRCA2 and the other less common high-risk genes are estimated to account for not more than 20 % of the familial risk [28, 29]. Since moderate risk genes are estimated to account for approximately 5 % and low risk genes for another 14 % of familial breast cancer risk, the genetic background in familial breast cancer still remains unclear to a large extent [24, 30].

**Fig. 1:**
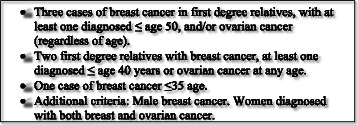
Swedish Breast Cancer Group BRCA 1 and BRCA 2 screening criteria

In summary, these findings suggest that there may be other breast cancer syndromes yet to be identified. This is supported by a Swedish study from 2007, by von Wachenfeldt et al., who investigated tumour spectrum in 803 Swedish families with hereditary breast cancer suggesting that breast cancer and endometrial cancer could constitute a new syndrome [31]. The Department of Clinical Genetics at Karolinska University Hospital has a long history of genetic counselling in breast cancer families in the Stockholm County. In all families fulfilling national screening criteria, pathogenic mutations in BRCA1 and 2 are ruled out (Fig. 1). Other previously identified risk genes are analysed only if family pattern indicates a specific syndrome. Accordingly, we now have a large cohort of consecutive families with hereditary breast cancer where the underlying cause is unclear. Our aim in this study was to search for new breast cancer syndromes in that cohort of non-BRCA families. We were particularly interested in carrying out a follow up on the results of a putative breast and endometrial cancer syndrome.

## Methods

All families with hereditary breast cancer who were subjected to genetic screening of BRCA1 and BRCA2 from February 2000 to January 2012 in the county of Stockholm, Sweden, were eligible for the study. This time period was chosen in order to avoid risking an overlap with the von Wachenfeldt study [31]. Probands in these families had either been referred or had contacted Karolinska University Hospital themselves for genetic counselling. Pedigrees were constructed for each family and the genetic counsellor verified cancer diagnosis through medical records, information from the Swedish cancer registry and death certificates when possible and if it was considered of importance in the individual family. Families with disease causing variants in the breast cancer genes BRCA1 or BRCA2 or other previously identified cancer syndromes were excluded. All genetic testing was performed in the same laboratory at the Department of Oncology at Lund University Hospital. For BRCA mutation analysis, denaturing high performance liquid chromatography (DHPLC) was used as the screening tool between 2000 and 2005. In addition to DHPLC, from 2006 to 2010 multiple ligation-dependent probe amplification (MLPA) was performed to exclude larger genomic rearrangements. Together, the DHPLC and MLPA have a stated sensitivity of 95 %. For cases before 2006, blood samples were reanalysed using MLPA when the technique was introduced. For samples from the year 2010 and later, analysis was performed using Next Generation Sequencing with a sensitivity of over 95 %.

**Fig. 2:**
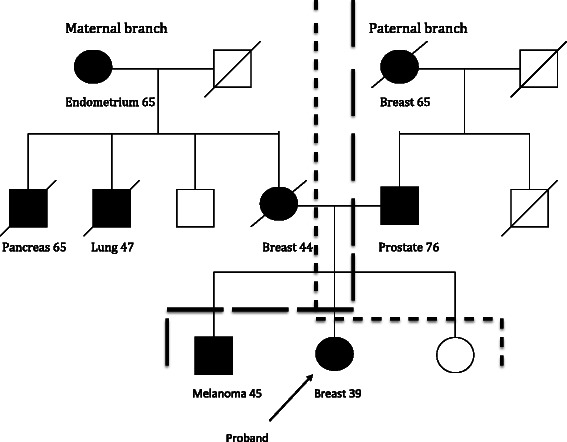
Maternal and paternal branches are considered as two families although all diagnosis are registrered only once. Maternal branch marked with *short lines* and paternal branch marked with *long lines*

Pedigrees from the non-BRCA families were examined and included if they contained at least two cases of breast cancer and one case of any other type of cancer in first or second degree relatives or first cousins on either maternal or paternal branch of the family. Kinship was always related to the index patient. If these criteria were fulfilled in both the maternal and the paternal branches, diagnoses from both branches were registered, however, each individual cancer diagnosis could only be included once (Fig. 2). All diagnoses other than breast cancer in first or second-degree relatives or cousins were registered as well as age at onset when data was available.

Distribution of cancer diagnoses in the data was compared to the distribution of cancer diagnoses in the general Swedish population. Since selection of cases was made on the basis of breast cancer diagnoses only, diagnoses other than breast cancer were used in the comparison. Data of cancer diagnoses in the Swedish population was obtained from the National Board of Health and Welfare (Socialstyrelsen). Indirect standardisation was used here to adjust the data from the Swedish population to that of the relatives with cancer diagnoses with regard to gender and age. Age was categorised into 5-year intervals. For relatives with missing data on gender or age, the method data Missing Completely At Random [32] was assumed. Cancer cases in the relatives were assumed to be independent of each other. Confidence intervals were calculated separately for each cancer diagnosis, using a binomial distribution. The number of cases was then transformed into proportion of cases by dividing by the total number of observed cases. Population data were assumed to reflect a true distribution, and were used as reference values. Two reference years were chosen, 1970 and 2010. A cancer diagnosis was regarded as overrepresented in the relatives of the breast cancer patients if the confidence interval was above both population reference values. All statistical analyses were performed in R (R Core Team, 2012). Data entry was performed in EpiData (Lauritsen).

## Results

**Fig. 3:**
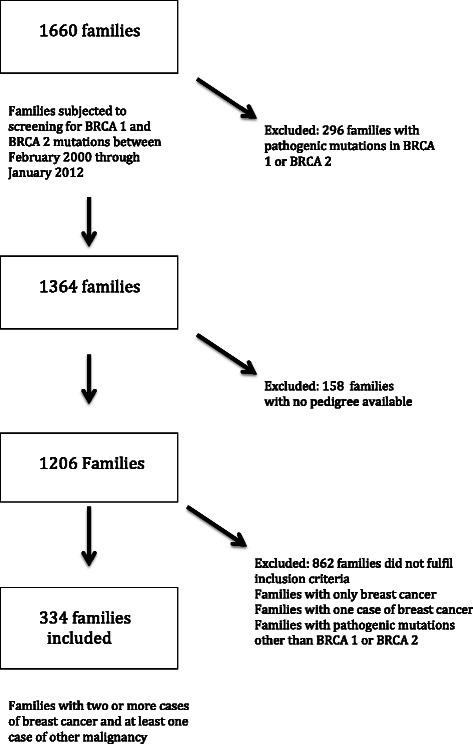
Flowchart illustrating inclusion and exclusion of study families

Pedigrees from 334 non-BRCA families remained for inclusion in this study after excluding BRCA positive families, families who did not fulfill inclusion criteria and families with no pedigree available (Fig. 3). Within these families we found 707 cases of cancer other than breast cancer, equaling 2.2 cases per family. 54 % of the cancer diagnoses were verified histologically from the Swedish cancer registry, medical records or death certificates. In 33 families, diagnoses were registered from both maternal and paternal branches (Fig. 2).

The proportions of different cancer types in the study families were compared to the proportion of cancer types in the Swedish cancer population in 1970 and 2010; the method used is described above in material and methods. We found an increased incidence of endometrial cancer representing a proportion of 6.36 % (CI 4.67–8.2) in the study families compared to the incidence in the general population in the reference years 1970 and 2010 (3.07 % in 1970, 2.64 % in 2010). Also liver cancer was observed in a higher proportion than expected (2.12 %; CI 1.13–3.25, 0.28 % in 1970 and 0.13 % in 2010) (Table 1). Moreover, several BRCA-related cancer types were overrepresented in the study population: ovarian cancer in 10.33 % (CI 8.2–12.59, 4.51 % in 1970, 1.93 % in 2010), prostate cancer in 14.57 % (CI 12.02–17.26, 6.98 % in 1970, 11.95 % in 2010) and pancreatic cancer in 4.67 % (CI 3.11–6.22, 2.79 % in 1970, 1.54 % in 2010). In contrast, low incidence cancer types such as small intestine cancer, thyroid cancer and other endocrine cancers were present in a lower proportion in the study families compared to the reference population. Several rare cancer types like genital cancers were not represented at all in the study population (Table 1).

**Table 1:** Proportion of different cancer types in non-BRCA families

Cancer site	Observed number	Proportion [%]	LL 95 %	UL 95 %	Proportion [%] in Sweden 1970	Proportion [%] in Sweden 2010	Reference outside CI
Prostate	103	14.57	12.02	17.26	6.98	11.95	CI above reference
Ovary/Fallopian tube	73	10.33	8.2	12.59	4.51	1.93	CI above reference
Colon	47	6.65	4.81	8.49	7.75	7.21	No
Lung	45	6.36	4.67	8.2	5.5	5.53	No
Uterus	45	6.36	4.67	8.2	3.07	2.64	CI above reference
Malignant melanoma	35	4.95	3.39	6.65	4.28	9.04	No
Brain and nervous system	34	4.81	3.25	6.51	7.66	6.12	No
Pancreas	33	4.67	3.11	6.22	2.79	1.54	CI above reference
Uterine cervix	33	4.67	3.11	6.22	4.85	2.98	No
Stomach	28	3.96	2.55	5.52	6.51	1.49	No
Abdomen UNS	25	3.54	2.26	4.95	0	0	CI above reference
Urinary tract	24	3.39	2.12	4.81	3.59	3.72	No
Kidney	23	3.25	1.98	4.67	4.13	2.28	No
Leukemia	23	3.25	1.98	4.67	4.55	5.25	No
Rectum and anus	21	2.97	1.84	4.24	3.73	3.72	No
Lymphoma	21	2.97	1.84	4.24	5.75	6.28	CI below reference
Liver	15	2.12	1.13	3.25	0.28	0.13	CI above reference
Biliary tract	13	1.84	0.99	2.83	2.85	1.47	No
Skin	13	1.84	0.99	2.83	2.4	7.92	No
Oesophagus	9	1.27	0.57	2.12	0.76	0.59	No
Connective tissue	8	1.13	0.42	1.98	1.43	1.15	No
Multiple myeloma	8	1.13	0.42	1.98	1.13	1.05	No
Testis	6	0.85	0.28	1.56	1.52	3.27	No
Thyroid	5	0.71	0.14	1.41	1.97	2.72	CI below reference
Mouth	4	0.57	0.14	1.13	0.91	1.2	No
Endocrine	4	0.57	0.14	1.13	2.31	2.01	CI below reference
Lip	2	0.28	0	0.71	0.65	0.18	No
Salivary gland	2	0.28	0	0.71	0.54	0.47	No
Larynx	2	0.28	0	0.71	0.56	0.29	No
Unspecified	2	0.28	0	0.71	3.29	2.36	CI below reference
Small intestine	1	0.14	0	0.42	0.43	0.47	CI below reference
Tongue	0	0	0	0	0.22	0.45	CI below reference
Peritoneum	0	0	0	0	0.03	0.16	CI below reference
Middle ear	0	0	0	0	0.38	0.17	CI below reference
Mediastinum	0	0	0	0	0.01	0.07	CI below reference
Placenta	0	0	0	0	0.11	0.05	CI below reference
Female genital	0	0	0	0	0.53	0.42	CI below reference
Male genital	0	0	0	0	0.24	0.14	CI below reference
Eye	0	0	0	0	0.55	0.45	CI below reference
Skeleton	0	0	0	0	0.82	0.57	CI below reference
Polycytemia vera	0	0	0	0	0.37	0.26	CI below reference
Myelofibrosis	0	0	0	0	0.03	0.32	CI below reference

## Discussion

The main finding of the present study is that endometrial cancer was overrepresented in the study population of families with hereditary non-BRCA breast cancer compared to the reference population, supporting the earlier findings of a putative breast cancer and endometrial cancer syndrome [31]. A strength of the study, compared to von Wachenfeldt et al, is that pathogenic mutations in BRCA1 and BRCA2 have been excluded through standardised techniques in the index patient in all of the families. Subsequently, the study cohort is more likely to harbour other genetic risk factors. The methodology was otherwise similar to that used in the previous study. A potential familial association between endometrial cancer and breast cancer has been studied in other settings with differing results, though few of the studies are based on cohorts with familial clustering of breast cancer. Lynch et al. identified an excess of endometrial cancer in families with two or more cases of breast cancer [33]. In families with a history of endometrial cancer Andersson et al. found a significantly increased risk for breast cancer in first-degree relatives of women affected by bilateral breast cancer [34]. In a prospective cohort study, Kazerouni et al. found no association between familial breast cancer and endometrial cancer. However the risk of endometrial cancer in women with a first-degree relative with bilateral breast cancer was increased by 40 % though that increase was not statistically significant [35]. Bilateral breast cancer and also the number of cases and age at onset are markers of familial breast cancer and this indicates that the elevated risk of cancer is influenced by a genetic mechanism [36, 37].

Given these points, the results from Kazerouni et al. and Andersson et al. are of great interest. Contrary to this, Tzortzatos et al. found no association between endometrial cancer in a study on tumour spectrum in a cohort of consecutive cases of endometrial cancer and breast cancer [38]. Pazzerini et al. investigated family history of endometrial cancer in a case control cohort of women with breast cancer and found no association [39]. Regardless of family history, an association between breast and endometrial cancer in the same individual, double primaries, has been established in several studies which provides evidence for an aetiological association [35, 38]. The two tumour types also share hormonal and reproductive risk factors such as obesity, nulliparity and exogenous oestrogenes [40, 41]. Shared environmental factors and/or genetic risk factors could be behind the pathogenesis of theses cancers [40, 41]. Younger women are especially at risk of developing serous carcinoma, a subtype of endometrial cancer independent of oestrogen and associated with risk of breast cancer, which raises the possibility of predisposing genetic factors [42]. Two autosomal dominant genetic disorders cause increased risk of developing endometrial cancer. One of them, Cowden syndrome, caused by germ-line mutations in PTEN also confers increased risk of developing breast cancer [10]. Hereditary non-polyposis colorectal cancer (HNPCC) or Lynch syndrome is caused by a defective mismatch repair system and causes increased risk of developing endometrial cancer. Evidence for an association with breast cancer risk has not been shown even though some role of mismatch repair genes in breast cancer development in these families has been suggested [43, 44]. In our cohort, all pedigrees were assessed by genetic counsellors and in relevant cases specific high risk syndromes were ruled out by genetic screening. It is therefore unlikely that families with either Cowden or Lynch syndrome are included in the study. Tamoxifen treatment is used in adjuvant breast cancer treatment and has a carcinogenic effect on the endometrium in postmenopausal women and doubles the risk of endometrial cancer and the risk increases with longer duration [45–47]. In our cohort, four women were affected by endometrial cancer between 1 and 18 years after a breast cancer diagnosis. One of these women with breast cancer was diagnosed before Tamoxifen was introduced onto the market. Two of the women were affected at a young age in the 1980s; Tamoxifen was not used in premenopausal women in Sweden at that time. Thereby, the development of endometrial cancer subsequent to breast cancer cannot be attributed to Tamoxifen use in these women. The fourth case was a premenopausal woman who received 2 years of adjuvant Goserelin and Tamoxifen.

However, there are several issues that need to be discussed. In general, the awareness of hereditary breast and ovarian cancer syndrome is good among clinicians. Therefore, there is a possibility that the large proportion of ovarian cancer could be due to selection bias. Selection bias could also partly explain the excess of other types of malignancies in the study group since having any cancer diagnosis other than breast cancer might result in a referral for genetic counselling. Nonetheless, not all cancer types were overrepresented in the families. One hundred fifty eight pedigrees could not be obtained but it is unlikely that that would cause any kind of bias. A random sample from medical records for the families with missing pedigrees showed that most of these families did not fulfil inclusion criteria and that a pedigree was not constructed due to only one affected woman at a young age.

In the study, all diagnoses in first or second-degree relatives or first cousins were included regardless of whether they were verified through medical records, death certificates or not. We decided to include unverified diagnoses so as not to lose potentially important information and since the accuracy of information on family history of cancer in close relatives gathered from probands is generally high [48, 49]. Nevertheless, in order to reduce the risk for misclassifications of malignancies as well as over- or underreporting due to recall bias, malignancies in third-degree relatives were included only in first cousins and not in older generations. Distinguishing ovarian cancers from endometrial or cervical cancers is important in order to correctly identify all families with hereditary breast and ovarian cancer syndrome since they can be offered genetic testing. As a result, counsellors put great efforts into verifying cancer diagnoses histologically in families with a history of gynaecological tumours and the majority of gynaecological cancers (e.g. 78 % of cases of endometrial cancer) were verified through medical records or death certificates. In contrast, malignancies such as lung cancer and liver cancer were verified to a smaller degree and the overrepresentation of liver cancer may partly be an effect of misclassification of metastatic liver disease. As for rare tumour types it is difficult to draw any conclusions since the statistical method compares proportions not incidence (a higher incidence in the study population may go undetected since proportions will be dominated by the most common cancer types).

We also found an overrepresentation of prostate, ovarian and pancreatic cancer. Germ line mutations of BRCA2 are associated with not only an increased risk of breast cancer and ovarian cancer but also with prostate cancer and pancreatic cancer. The result therefore suggests a BRCA2-like syndrome in non-BRCA families, bearing in mind that most identified moderate risk genes functionally interact with BRCA1 and BRCA2. However, it has been considered unlikely that more risk genes with effects similar to BRCA1 and BRCA2 exist. Extensive search with linkage studies has failed to identify further high penetrance risk genes affecting breast cancer susceptibility. Nevertheless, Antoniou et al. recently presented data on breast cancer risk attributed to PALB2 mutations at the same level as BRCA2 mutations [19]. In addition, the risk was higher for PALB2 mutation carriers with a family history of breast cancer compared to no family history suggesting that the risk is influenced by other genetic factors and/or environmental factors. The PALB2 protein interacts with BRCA2 in homologous recombination and double strand break repair. As with BRCA2, germ line mutations of PALB2 confer increased risk of breast cancer as well as ovarian and pancreatic cancer [20, 21]. Since PALB2 mutations are rare, the contribution to the increased risk for these tumour types should be limited in the study cohort. Nevertheless, PALB2 mutations and pathogenic mutations in other genes involved in the same pathway as BRCA2 could explain a minor part of the excess of pancreatic and ovarian cancer. It has also been suggested that a polygenic model in which a large number of moderate and/or low risk genes combined has multiplicative effects on risk and could partially explain the genetic background in familial breast cancer [29]. Since the sensitivity of BRCA mutation testing is 95 %, a part of the excess of ovarian, pancreatic and prostate cancers could reflect false negative screening in the research cohort. As mentioned above, a part of at least the excess of ovarian cancer could be a result of selection bias.

## Conclusions

To summarise, we found an overrepresentation of endometrial cancer in our cohort of hereditary non-BRCA families, which supports earlier findings that breast and endometrial cancer may constitute a breast cancer syndrome. Since results from studies are divergent this issue needs to be resolved by further studies preferably on cohorts with two close relatives or more affected by breast cancer or bilateral breast cancer. The conflicting results could be due to methodology since the association may only be evident in families with a strong pattern of breast cancer susceptibility. Identifying new breast cancer syndromes is of importance to reach more women at increased risk of cancer with preventive programmes. It is also a first step towards detection of new susceptibility genes.

### Ethical statement

The study was approved by the Ethics Committee of Karolinska Institutet/ Karolinska University Hospital (DNR).
